# Endothelial Glycocalyx in Cerebral Infarction After Endovascular Treatment in Patients With Intracranial Artery Stenosis

**DOI:** 10.1111/cns.70545

**Published:** 2025-08-13

**Authors:** Fangfang Zhao, Tao Wang, Jichang Luo, Haoyuan Gao, Yangmin Zheng, Rongliang Wang, Junfen Fan, Haiping Zhao, Ziping Han, Yumin Luo, Liqun Jiao

**Affiliations:** ^1^ Institute of Cerebrovascular Disease Research and Department of Neurology Xuanwu Hospital of Capital Medical University Beijing China; ^2^ Department of Neurosurgery Xuanwu Hospital, Capital Medical University Beijing China; ^3^ Department of Cardiology The Second Affiliated Hospital of Shandong First Medical University Taian China; ^4^ Beijing Geriatric Medical Research Center and Beijing Key Laboratory of Translational Medicine for Cerebrovascular Diseases Beijing China; ^5^ Beijing Institute for Brain Disorders, Capital Medical University Beijing China

**Keywords:** endovascular treatment, glycocalyx, hyaluronic acid, intracranial artery stenosis

## Abstract

**Objective:**

To examine whether plasma glycocalyx levels in patients with severe intracranial arterial stenosis (ICAS) are associated with the prediction of new cerebral infarctions following endovascular treatment.

**Methods:**

105 patients with ICAS who underwent endovascular treatment were enrolled from September 2020 to June 2021. Plasma glycocalyx components were detected. The condition of the cerebral artery was obtained by high‐resolution nuclear magnetic resonance, and risk factors for new cerebral infarction postendovascular treatment in patients with ICAS were analyzed using both univariate and multivariate analyses. The results were presented in a nomogram risk model and evaluated.

**Results:**

The plasma glycocalyx in patients with ICAS exhibited significant differences compared to healthy individuals (*p* < 0.05). Multivariate analysis revealed eccentricity (OR = 3.599, 95% CI: 1.168–12.478, *p* = 0.032) and hyaluronic acid (OR = 5.542, 95% CI: 1.545–23.573, *p* = 0.013) as risk factors (*p* < 0.05). The risk model encompassing eccentricity, Remodeling index, blood glucose, hyaluronic acid (HA), and age was presented as a Nomogram, with a C‐index of 0.810 (95% CI, 0.729–0.890). To further analyze the risk factors affecting HA plasma concentration, the multivariate analysis showed that fibrinogen and hemoglobin A1c (HbA1C) significantly affected changes in HA.

**Conclusion:**

We found that HA is a critical factor in predicting adverse events of cerebral infarction after endovascular treatment in patients with ICAS. Additionally, HbA1C and fibrinogen were identified as factors influencing HA changes.

**Trial Registration:**

ClinicalTrials.gov Identifier: NCT01994161

## Graphical abstract

The predictive role of Glycocalyx in cerebral infarction after endovascular treatment in patients with cerebral artery stenosis.

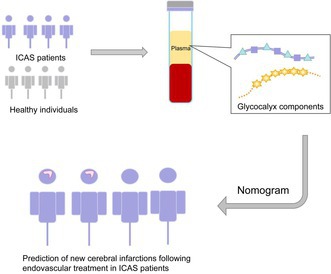

## 1 Introduction

Intracranial atherosclerotic stenosis (ICAS) is one of the most common causes of stroke globally, with high morbidity and mortality rates [1, 2]. It is particularly prevalent in Asian populations, making it the most common cause of stroke within this demographic [2, 3]. Contemporary treatments for ICAS include drug therapy and endovascular therapy. The SAMMPRIS study, however, indicated that endovascular therapy does not outperform medical therapy [4]. Furthermore, recent results from the China Angioplasty and Stenting for Symptomatic Intracranial Severe Stenosis (CASSISS) study revealed no significant differences between medical and endovascular treatments for patients with ICAS [5]. A history of endovascular therapy may increase the likelihood of a new cerebral infarction rapidly. A study has shown that the incidence of new cerebral infarctions following endovascular treatment in patients with ICAS is 55.1% [6]. Even with strict control of vascular risk factors, the risk of silent infarction can increase by two to three times [7]. Therefore, the safety and effectiveness of endovascular treatment for ICAS need to be further investigated, and it is crucial to elucidate the risk of new cerebral infarction after endovascular treatment.

Endothelial glycocalyx (EG) refers to glycosaminoglycans (GAGs) and proteoglycans (PGs) that cover the luminal surface of endothelial cells, exhibit gel‐like properties, line arterial luminal sides, and interact with flowing blood [8]. PGs include Syndecan‐1, Syndecan‐2, Syndecan‐3, Syndecan‐4, Glypican (GPC‐1), and Biglycan. The GAG chains include heparan sulfate (HS), chondroitin sulfate (CS), keratin sulfate (KS) binding to PGs, and hyaluronic acid (HA) which does not bind to PGs [9]. EG protects vascular endothelial cells against atherosclerosis by limiting lipid deposition and inhibiting inflammatory responses [10, 11]. EG loss [12] and associated lipid deposition [13] are correlated with the progression or stability of arterial plaques. Therefore, modifications in EG molecules are likely important contributors to plaque instability. The prevalent mechanism of new cerebral infarction following ICAS endovascular treatment is plaque instability and arterial–arterial embolism [6]. Therefore, we hypothesized that alterations in EG molecules in the intracranial arterial wall are an important cause of new cerebral infarction after ICAS endovascular treatment. This study aimed to identify additional risk factors influencing new cerebral infarctions following endovascular treatment in patients with ICAS, to offer new insights for mitigating adverse events post‐endovascular treatment in these patients.

## 2 Materials and Methods

### 2.1 Patients Selection

**FIGURE 1:**
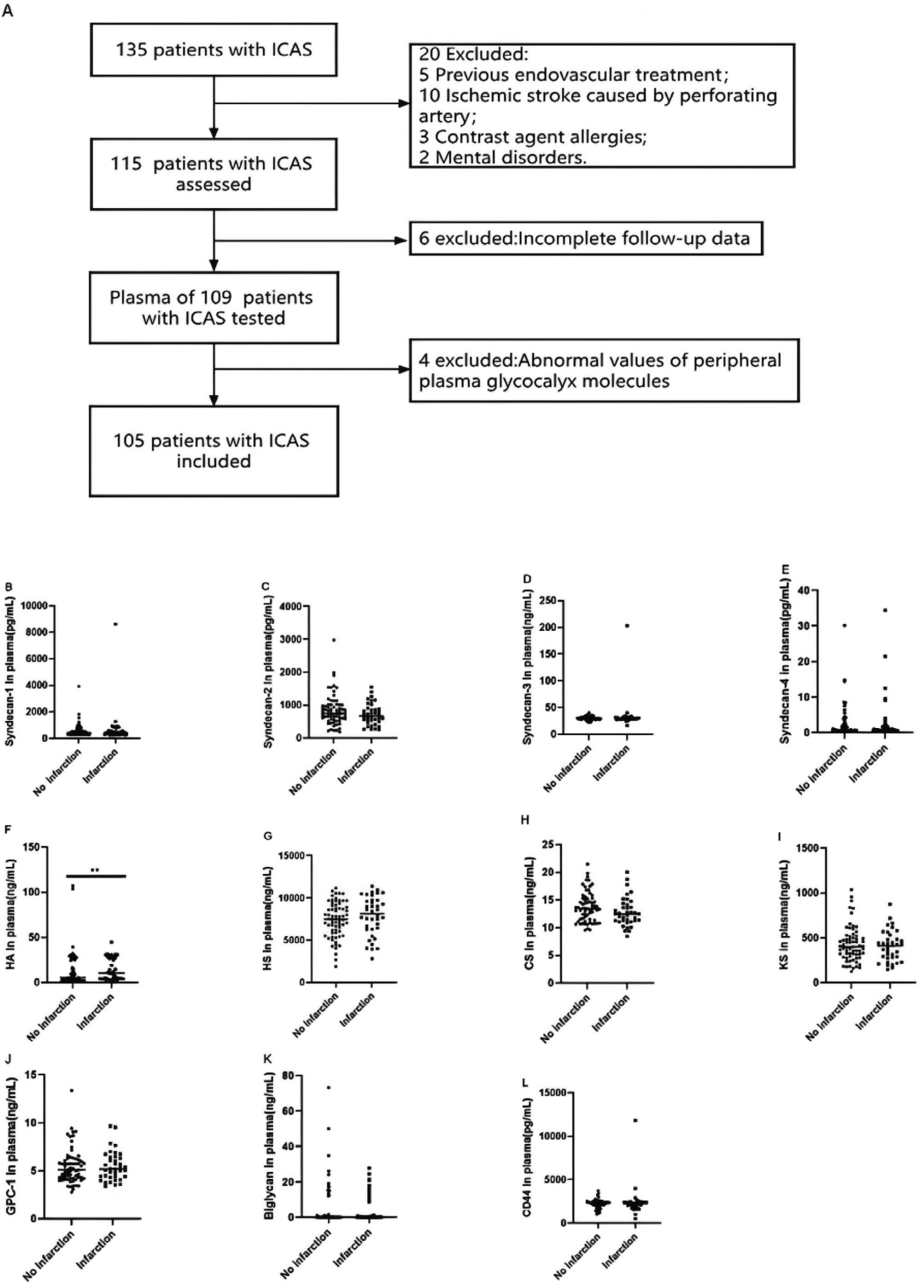
Study flow diagram and comparison of plasma EG levels between ICAS patients with infarction and no infarction. (A) Study flow diagram; (B), Plasma level of Syndecan‐1, Syndecan‐2, Syndecan‐3, Syndecan‐4, HA, HS, CS, KS, GPC‐1, Biglycan, CD44. *N* = 105 for patients with ICAS. **p < 0.001, *p < 0.05.

This retrospective observational study included patients admitted to Xuanwu Hospital of Capital University of Medical Sciences, based on data collected from September 2020 to June 2021. The study was approved by the Ethics Committee of Xuanwu Hospital, Capital Medical University (No. [2013] 004). All patients or their immediate family members provided written informed consent. Inclusion criteria comprised patients aged 18–80, with a TIA or nondisabling ischemic stroke, an mRS ≤ 2, severe stenosis (70%–99%) of the intracranial large artery supplying the ischemic event region, at least one stenotic artery being a major intracranial artery (internal carotid artery, vertebral artery, middle cerebral artery, or basilar artery), and vascular stenosis confirmed according to the Warfarin‐aspirin symptomatic intracranial disease study (WASID) criteria [14]. All patients underwent HR‐MRI before intervention, and DWI sequences were performed 72 h pre‐ and postsurgery. The study excluded patients with major cerebral infarction or intracranial hemorrhage on CT or MRI scans, nonarteriosclerosis‐related intracranial artery stenosis, cerebral infarction due to basal ganglia or perforating brain stem arteries, severe blood diseases, tumors, mental illnesses, epilepsy, pregnancy, or those refusing to participate in the clinical trial. Ultimately, 105 patients with ICAS (Figure 1A) were included in our study.

### 2.2 Clinical Data Collection

Collected clinical data included demographic characteristics (age, sex, BMI), smoking and drinking history, past medical history, high‐resolution nuclear magnetic results, biological measures, and EG‐related molecular detection. The primary endpoint of this study was the occurrence of a new cerebral infarction on DWI within 72 h postendovascular treatment.

### 2.3 Molecular Detection of EG

We collected patient plasma samples in EDTA tubes within 3 days of admission before intracranial artery stenting. Samples were centrifuged at 1000 g for 10 min, and the separated plasma was stored at −80°C until testing, with single tube use to avoid repeated freeze–thaw cycles. Enzyme‐linked immunosorbent assay (ELISA) kits were employed to quantify the concentrations of Syndecan‐1 (R&D, DY2780), Syndecan‐2 (Cusabio, CSB‐EL020889HU), Syndecan‐3 (Shanghai Enzyme‐linked Biotechnology Co. Ltd., ml027594), Syndecan‐4 (Cusabio, CSB‐EL020891HU), Glypican (Cusabio, CSB‐EL009703HU), Biglycan (Cusabio, CSB‐EL002683HU), CD44 (Shanghai Enzyme‐linked Biotechnology Co. Ltd., ml057407), HA (Cusabio, CSB‐E04805h), HS (Cusabio, CSB‐E09585h), KS (Cloud‐Clone, CEA590Hu), and CS (abbexa, abx052801). All tests followed the manufacturers' instructions. The assay instrument was a Bio‐Tek Elx800 microplate reader.

### 2.4 Magnetic Resonance Imaging Analysis

All eligible patients were examined using a 3.0T magnetic resonance imaging (MRI) system (MAGNETOM Avanto; Siemens Healthineers). Two experienced physicians analyzed the imaging readings. The Remodeling index was defined as: [VA_MLN_/VA_REF_], where VA_MLN_ is the vessel area at the most stenotic site and VA_REF_ is the reference vessel area. Positive remodeling was indicated by a Remodeling index > 1.05, whereas a Remodeling index ≤ 1.05 indicated nonpositive remodeling. Positive remodeling is more common in vulnerable plaques, leading to clinical symptoms. Contrast enhancement ratios were measured at the point of maximum lumen narrowing. The enhancement ratio was computed as [plaque signal (after contrast)/gray matter signal (after contrast)]/[plaque signal (before contrast)/gray matter signal (before contrast)] × 100% [15]. The enhancement ratio was categorized into: no reinforcement, level 1 reinforcement, level 2 reinforcement (no enhancement: enhancement similar to or less than the enhancement of the intracranial arterial wall without plaque; Level 1: enhancement greater than grade 0 but less than the pituitary funnel; Level 2: enhancement similar to or greater than that of the pituitary funnel). The degree of plaque enhancement reflects the degree of inflammatory activity.

### 2.5 Procedure and Analysis of Endovascular Treatment

The CASSISS study was used as the reference for endovascular treatment, which is performed by experienced neurosurgeons, including primary angioplasty, balloon and self‐expandable stent placement (Apollo) and Gateway‐Wingspan systems. Endovascular treatment was decided by the operator according to the characteristics of the lesion and his own experience. All patients received a combination of aspirin (100 mg daily) and clopidogrel (75 mg daily) preoperatively, or a loading dose of aspirin and clopidogrel (300 mg each) starting 1 day preoperatively.

### 2.6 Statistical Analysis

Statistical analysis was conducted using SPSS 24.0 and R language version 4.2.2. For count data that conformed to normal distribution, the mean (x̄) ± SD was used; if not, median (IQR) is used for categorical data, and frequency (%) is used for categorical data. For normally distributed count data, a *t*‐test or analysis of variance was used for group comparisons, while nonparametric tests were used for nonnormally distributed count data. Chi‐squared tests were applied for categorical data. Correlation analysis was performed using either Spearman or Pearson correlation coefficients. For independent variable screening with cerebral infarction as the outcome dependent variable, the method of LASSO regression was employed. The independent variables obtained via LASSO regression were incorporated into nomograms, and the models were evaluated using receiver operating characteristic curve (ROC) and decision curve analyses (DCA). Binary logistic regression models were utilized to determine the risk factors. A *p* value < 0.05 was considered statistically significant. The R packages used include glmnet, rms, readr, Hmisc, forestplot, readxl, nomogram, pROC, and rmda.

## 3 Results

### 3.1 Demographics

From September 2020 to June 2021, a total of 105 ICAS patients were enrolled in the study. The average age of ICAS patients was 59.79 years, 79% of whom were male. The research results show a history of diabetes (52.6%; *p* = 0.032), eccentricity (81.6; *p* = 0.015), remodeling index > 1.05 (36.8%; *p* = 0.018), and hyperglycemia (median 6.66; IQR [5.52, 9.13]; *p* = 0.002) were more likely to have postoperative cerebral infarction (Table 1).

**TABLE 1:** Comparison of baseline data in patients with intracranial arterial stenosis.

	Postoperative cerebral infarction			
	All (105)	No (*n* = 67)	Yes (*n* = 38)	*p*
**Demographic characteristics [*n*(%) OR median (IQR) OR x ± s]**				
Age, years (x ± s)	59.79 ± 8.24	58.67 ± 8.29	61.76 ± 7.87	0.064
BMI (kg/m^2^)	26.07 ± 3.18	25.86 ± 3.43	26.43 ± 2.70	0.384
Male, *n* (%)	83 (79.0)	52 (77.6)	31 (81.6)	0.631
**Medical history [*n* (%)]**				
Smoking, *n* (%)	53 (50.5)	33 (49.3)	20 (52.6)	0.739
Drinking, *n* (%)	40 (38.1)	26 (38.8)	14 (36.8)	0.842
CVD, *n* (%)	5 (4.8)	3 (4.5)	2	0.856
Hypertension, *n* (%)	83 (79.0)	50 (74.6)	33 (86.8)	0.139
Diabetes, *n* (%)	41 (39.0)	21 (31.3)	20 (52.6)	0.032
Hyperlipidemia, *n* (%)	44 (41.9)	25 (37.3)	19 (50.0)	0.205
**Preoperative NIHSS and mRs score**				
Preoperative NIHSS score	0.58 ± 1.39	0.55 ± 1.46	0.63 ± 1.28	0.781
Preoperative mRs score	0.44 ± 0.87	0.46 ± 0.82	0.39 ± 0.95	0.701
**Biological measures[median (IQR) OR x ± s]**				
WBC (×109/L)	8.06 ± 13.80	8.79 ± 17.24	6.77 ± 1.33	0.473
PLT (×109/L)	221.97 ± 63.74	217.45 ± 68.50	229.95 ± 54.29	0.337
Hb (g/L)	139.46 ± 14.64	138.22 ± 15.07	141.63 ± 13.79	0.254
Neutrophil/Lymphocyte	3.01 ± 1.93	2.88 ± 2.06	3.25 ± 1.67	0.34
ALT (IU/L)	35.01 ± 25.41	35.31 ± 23.78	34.47 ± 28.39	0.872
AST (IU/L)	26.04 ± 10.65	26.22 ± 9.66	25.71 ± 12.34	0.814
HDL (mmol/L)	0.93 ± 0.23	0.94 ± 0.23	0.92 ± 0.23	0.783
LDL (mmol/L)	1.84 ± 0.69	1.79 ± 0.60	1.93 ± 0.82	0.322
TG (mmol/L)	1.02 [0.76, 1.45]	0.91 [0.74, 1.44]	1.12 [0.82, 1.50]	0.141
TC (mmol/L)	3.41 ± 1.00	3.30 ± 0.94	3.61 ± 1.09	0.134
Blood glucose (mmol/L)	5.96 [4.96, 7.70]	5.46 [4.73, 6.46]	6.66 [5.52, 9.13]	0.002
hsCRP (mg/L)	4.59 ± 10.82	4.69 ± 12.78	4.40 ± 6.15	0.893
ApoA (g/L)	0.97 (0.15)	0.96 (0.16)	0.99 (0.14)	0.418
ApoB (g/L)	0.72 (0.21)	0.70 (0.20)	0.76 (0.24)	0.223
*High‐resolution nuclear magnetic results*				
Vascular lesions [*n* (%)]				
**The_most_severe_stenosis_site**				
Anterior circulation	42 (40.0)	28 (41.8)	14 (36.8)	
Posterior circulation	63 (60.0)	39 (58.2)	24 (63.2)	0.619
Traffic_branch_opening, *n* (%)	64 (61.0)	41 (61.2)	23 (60.5)	0.946
Preoperative infarction, *n* (%)	52 (49.5)	31 (46.3)	21 (55.3)	0.376
Eccentric, *n* (%)	70 (66.7)	39 (58.2)	31 (81.6)	0.015
**Enhancement**				
No	10 (9.5)	5 (7.5)	5 (13.2)	0.634
Primary	49 (46.7)	32 (47.8)	17 (44.7)	
Secondary	46 (43.8)	30 (44.8)	16 (42.1)	
Remodeling index (> 1.05)	25 (23.8)	11 (16.4)	14 (36.8)	0.018
Area stenosis	78.44 ± 26.60	80.89 ± 14.61	74.10 ± 39.73	0.21
Plaque length (mm)	6.39 ± 4.04	5.98 ± 4.29	7.10 ± 3.49	0.173
Stenosis WASID (%)	77.53 ± 9.78	78.11 ± 10.70	76.50 ± 7.95	0.42

### 3.2 Predicting Risk Factors of Postoperative Cerebral Infarction by Predictive Models

**FIGURE 2:**
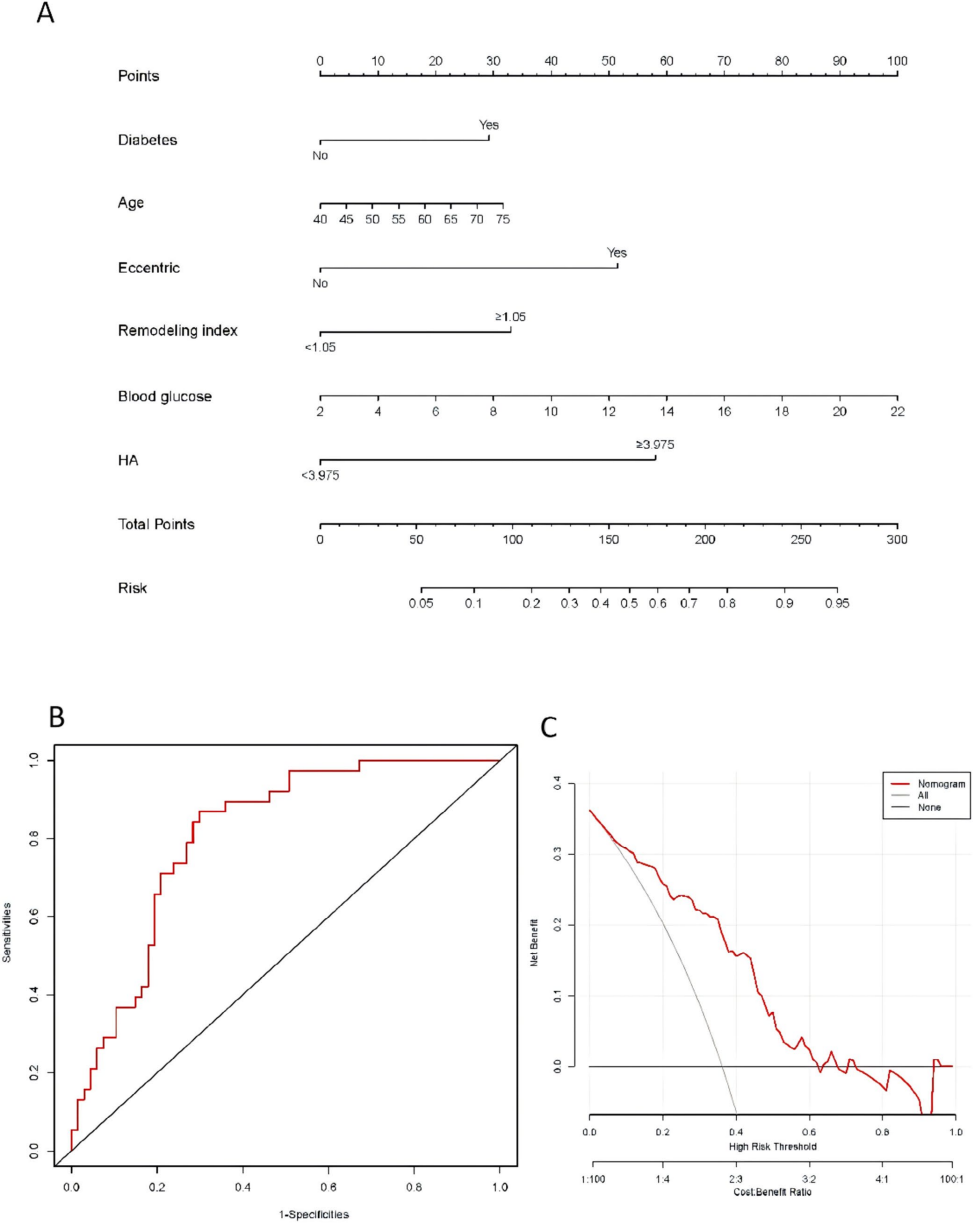
Nomogram and evaluation of Nomogram. (A) Nomogram, (B) ROC, (C) DCA.

**FIGURE 3:**
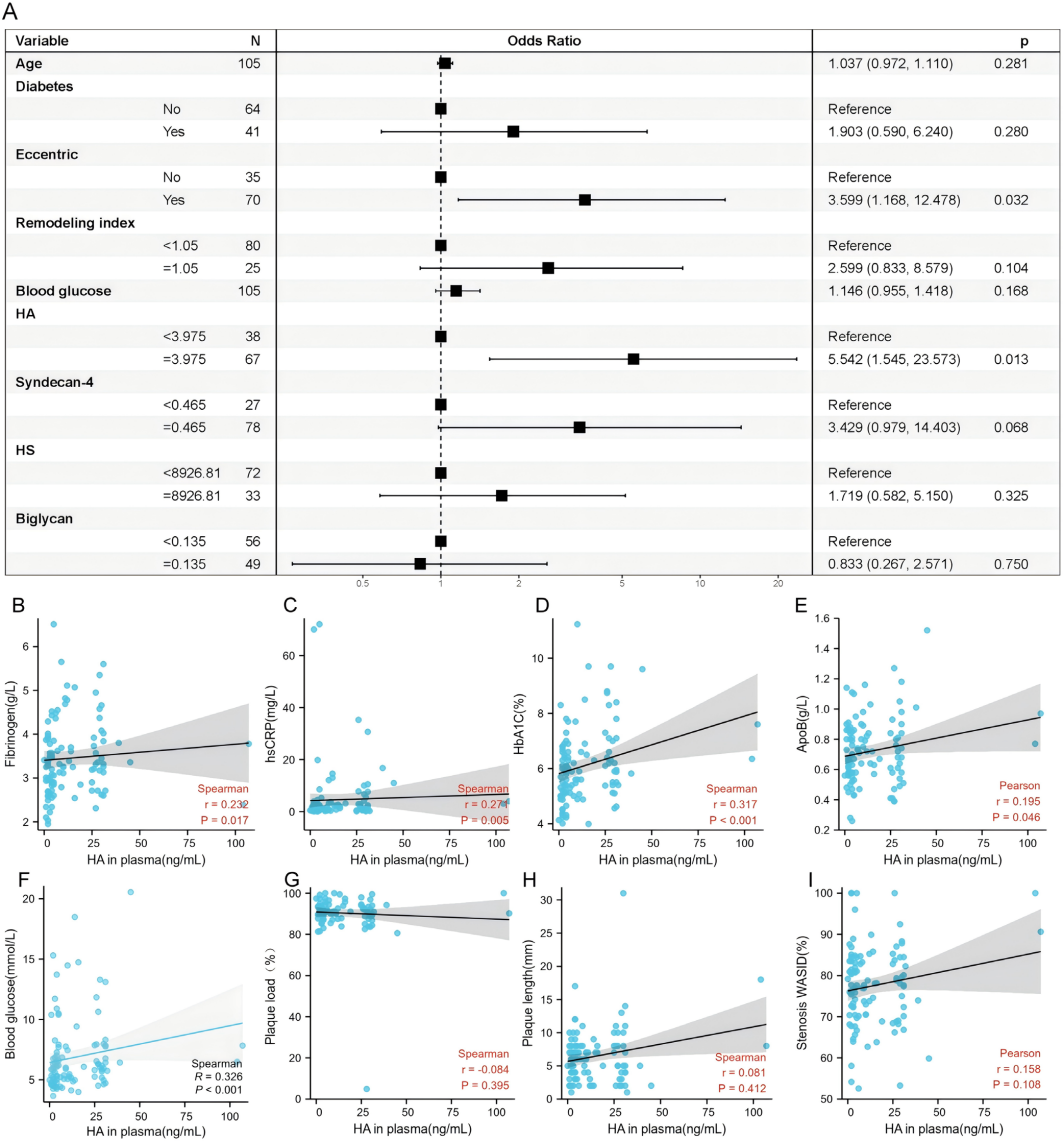
Forest plot and correlation analysis of HA plasma levels. (A) Forest plot of risk factors for new cerebral infarction after endovascular treatment in patients with ICAS; (B–I) the correlation of plasma HA level with fibrinogen, HbA1C, hsCRP, blood glucose, APOB, plaque area, plaque load, stenosis WASID.

Among all detected EG molecules, HA ≥ 3.975 ng/mL (81.6%; *p* = 0.004) is more prone to postoperative cerebral infarction (Figure 1F), the remaining molecules had no statistical significance (Figure 1B–E,G–L, *p* > 0.05). Of it, the optimal critical value of plasma HA level as an adverse outcome indicator was 3.975 ng/mL; sensitivity was 81.6%, specificity was 46.3% according to the ROC. The LASSO regression was utilized for screening the independent variables with the occurrence of postoperative cerebral infarction as the outcome variable. At the minimum lambda value of 0.08129065, four independent variables were identified that are significantly related to the occurrence of postoperative cerebral infarction, namely: eccentricity, Removal index, blood glucose, and HA. Another risk factor that might be related to the outcome variable, age, was also included. The prediction model of these screened risk factors for predicting the occurrence of postoperative cerebral infarction is presented as a nomogram (Figure 2A). The model's discrimination was calculated using the consistency index (C‐index), which resulted in a value of 0.810, and the 95% CI was 0.729–0.890. To further evaluate the prediction function of the nomogram, we also made an ROC curve (Figure 2B). The model exhibited good distinguishing ability and accuracy. The clinical utility of the nomogram was assessed using DCA. The DCA indicated that the nomogram has a high clinical application value in predicting the occurrence of postoperative cerebral infarction (Figure 2C). Binary logistic regression analysis was conducted with the presence or absence of postoperative cerebral infarction as the dependent variable, and variables with significant statistical significance in the univariate analysis (eccentricity, remodeling index, blood glucose, HA), and potentially clinically significant independent variables (Age, Diabetes, Syndecan‐4, HS, Biglycan) as independent variables. The results identified eccentricity (OR = 3.599, 95% CI: 1.168–12.478, *p* = 0.032) and HA (OR = 5.542, 95% CI: 1.545–23.573, *p* = 0.013) as risk factors impacting prognosis (*p* < 0.05, Figure 3A).

### 3.3 Predicting Risk Factors Affecting Plasma HA Levels

To further analyze the risk factors affecting HA plasma concentration, HA levels were then dichotomized (HA ≥ 3.975 ng/mL and HA < 3.975 ng/mL) using the critical points determined by the ROC. The results demonstrated that hypertension, hyperfibrinogenemia, hyperglycemia, high HbA1C, and high hsCRP significantly influenced the change of HA in plasma (Table 2, *p* < 0.05). Based on the results of univariate analysis, the relationship of plasma HA level with fibrinogen, HbA1C, hsCRP, blood glucose, APOB, and plaque‐related indices (such as plaque area, plaque load, and stenosis WASID) was examined. The findings showed that plasma HA levels had a significant positive correlation with fibrinogen, hsCRP, HbA1C, ApoB, and blood glucose (*p* < 0.05, Figure 3B–F). The data were divided into two groups using HA ≥ 3.975 as the cutoff value: HA ≥ 3.975 and HA < 3.975. A multivariate logistic regression analysis was performed with the elevation of plasma HA as the dependent variable and significant factors including hypertension, fibrinogen, HbA1C, hsCRP, and blood glucose as independent variables. The results indicated that fibrinogen and HbA1C were influential factors for changes in HA plasma concentrations (Table 3).

**TABLE 2:** Comparison of baseline data affecting peripheral plasma HA levels.

	HA (≥ 3.975 ng/mL)			
	All (105)	No (*n* = 38)	Yes (*n* = 67)	*p*
Age, years (x ± s)	59.79 ± 8.24	58.95 ± 8.62	60.27 ± 8.04	0.432
BMI (kg/m^2^)	26.07 ± 3.18	25.60 ± 3.10	26.33 ± 3.22	0.261
Male, *n* (%)	83 (79.0)	28 (73.7)	55 (82.1)	0.309
Smoking (%)	53 (50.5)	20 (52.6)	33 (49.3)	0.739
Drinking (%)	40 (38.1)	14 (36.8)	26 (38.8)	0.842
Hypertension (%)	83 (79.0)	26 (68.4)	57 (85.1)	0.044
Diabetes (%)	41 (39.0)	14 (36.8)	27 (40.3)	0.727
WBC (×109/L)	6.35 [5.34,7.67]	6.18 [5.26,7.66]	6.42 [5.44,7.63]	0.54
Neutrophil/lymphocyte	2.39 [1.86,3.69]	2.15 [1.75,3.87]	2.50 [2.01,3.67]	0.33
Fibrinogen (g/L)	3.32 [2.85,3.87]	3.16 [2.66,3.55]	3.36 [2.90,4.22]	0.025
HDL (mmol/L)	0.93 ± 0.23	0.95 ± 0.24	0.92 ± 0.23	0.654
LDL (mmol/L)	1.84 ± 0.69	1.72 ± 0.53	1.91 ± 0.76	0.168
TC (mmol/L)	3.41 ± 1.00	3.30 ± 0.90	3.48 ± 1.06	0.374
ApoA (g/L)	0.95 [0.86,1.06]	0.94 [0.85,1.01]	0.96 [0.88,1.06]	0.262
ApoB (g/L)	0.72 ± 0.21	0.67 ± 0.18	0.75 ± 0.23	0.054
HbA1C (%)	6.11 ± 1.353	5.56 ± 1.054	6.43 ± 1.410	0.001
Blood glucose (mmol/L)	5.96 [4.96,7.70]	5.01 [4.61,6.22]	6.31 [5.30,7.84]	0.001
hsCRP (mg/L)	1.33 [0.55,3.00]	1.00 [0.42,1.95]	2.01 [0.72,4.40]	0.008
Eccentricity	70 (66.7)	27 (71.1)	43 (64.2)	0.473
**Enhancement**				
No	10 (9.5)	2 (5.3)	8 (11.9)	0.519
Primary	49 (46.7)	18 (47.4)	31 (46.3)	
Secondary	46 (43.8)	18 (47.4)	28 (41.8)	
Remodeling_index (> 1.05)	25 (23.8)	10 (26.3)	15 (22.4)	0.65
Plaque load (%)	91.08 [89.49, 93.38]	91.57 [89.64, 93.03]	90.91 [88.79, 94.03]	0.805
Length (mm)	5.89 [3.94, 8.04]	5.05 [3.83, 6.94]	6.55 [4.04, 8.27]	0.105
Stenosis WASID (%)	77.68 [70.49, 83.51]	78.42 [72.80, 83.49]	77.64 [70.13, 83.47]	0.501

**TABLE 3:** Risk factors influencing the changes of HA in peripheral plasma.

	β	SE	Wald	Exp(β)	*p*
Hypertension	0.966	0.562	2.949	2.627	0.086
Fibrinogen	0.716	0.313	5.247	2.047	0.022
APOB	1.357	1.245	1.188	3.884	0.276
Blood glucose	−0.102	0.122	0.706	0.903	0.401
HbA1C	0.718	0.305	5.53	2.051	0.019
Constant	−7.149	1.907	14.05	0.001	0

## 4 Discussion

ICAS is one of the most common causes of stroke, with patients exhibiting severe (70%–99%) arterial stenosis being at a high risk of stroke recurrence [16]. Elevated levels of reactive oxygen species and other inflammatory mediators in patients with ICAS can lead to EG destruction. EG covers the healthy vascular endothelium and is an important component of the blood–brain barrier (BBB). As an important protector of BBB integrity [17], the EG is fragile and highly susceptible to ischemia and inflammation [18]. BBB dysfunction is associated with EG degradation. Early complications after endovascular therapy in patients with ICAS may be related to plaque shedding and/or reperfusion injury [4, 19]. However, both plaque shedding and reperfusion injury might be associated with the damage to the EG or BBB caused by EG degradation. Therefore, studying the molecular changes in EG is crucial for accurately predicting disease progression and the occurrence of cerebral infarction after endovascular treatment in patients with ICAS.

Our study showed that in the peripheral plasma of patients with severe ICAS, different EG molecules showed inconsistent trend levels of increase or decrease. Our findings suggest that EG molecules have variable levels of expression in advanced intracranial atherosclerosis (since our selected patients all had severe stenosis). To our knowledge, this is the first study to demonstrate alterations in plasma molecular levels of soluble EG components in ICAS patients. However, their exact role requires further study. Previous studies have also suggested that changes in glycocalyx molecules may be detected in peripheral plasma during acute or chronic disease states. Studies by Johansson and Hippensteel [20, 21] showed a 1–4‐fold increase in the plasma concentration of glycocalyx components in traumatic and inflammatory conditions. DellaValle et al. found that the levels of certain glycocalyx molecules in serum varied at different time points following acute cerebral infarction [22]. Additionally, Tang et al. found that plasma levels of HA significantly increased in patients with acute stroke, with plasma HA levels showing a U‐shaped correlation with clinical outcomes, suggesting that both high and low HA levels may lead to adverse outcomes [23]. Pathological studies have shown that the expression of glycocalyx at the atherosclerotic site varies at different stages of atherosclerosis. In early human atherosclerotic lesions, CS–lipoprotein complexes can be isolated [24]. As atherosclerosis progresses, the percentage of HA‐positive staining area in the lipid pool/necrotic core decreases from 33.4% in the early stage to 3.5% in the late stage [25]. Biglycan followed a similar pattern [25]. In conclusion, EG molecules may play a crucial role in intracranial atherosclerosis [26].

This study also investigated risk factors contributing to the incidence of adverse cerebral infarction events after ICAS. Eccentricity, HA, remodeling index, and blood glucose were identified as risk factors for cerebral infarction after intravascular ICAS therapy. These risk factors, in conjunction with age, which may have clinical significance, were utilized as independent variables to construct a nomogram, which demonstrated substantial clinical applicability. Intracranial atherosclerotic plaques are typically characterized by eccentricity wall thickening [27] and are more prone to progression [28]. The study found that, despite smaller volumes than those of centripetal plaques, eccentricity plaques can distort the vascular cavity's shape and create areas of high strain. This mechanical instability, caused by redistributed pressure within the cavity, makes the eccentricity plaque relatively unstable [29]. Consequently, after ICAS endovascular treatment, plaque shedding and subsequent cerebral infarction events may be more likely. Studies have shown that ICAS cases with positive remodeling are more prone to cerebral infarction than those without. Positive remodeling, an important vulnerability feature of high‐risk plaques, may cause alterations in the plaque's shoulder and internal stress, easily rupturing the fibrous cap and causing downstream embolism [30, 31]. Current research on intracranial arterial remodeling suggests that plaques with positive remodeling are closely related to unexplained ipsilateral cerebral embolism [31]. A speculated mechanism may be the presence of a larger lipid core volume concealed under the positively remodeled plaque. This might further facilitate the secretion of more metalloproteinases and the erosion of the fibrous cap, leading to thrombus [32] formation, which is more likely to lead to distal embolism. Additionally, persistent hyperglycemia and advanced age are also likely contributors to postoperative cerebral infarction in ICAS patients. Persistent hyperglycemia can induce endothelial EG damage [33], and cause microvascular lesions, leading to worse collateral circulation, which may promote postoperative cerebral infarction in ICAS patients. Studies have demonstrated that EG thickness decreases with age [34], making the BBB more susceptible to disruption, a potential cause of cerebral infarction after ICAS.

Interestingly, although plasma HA levels were lower in patients with ICAS than those in healthy individuals, higher plasma HA levels were more likely to cause new cerebral infarction following endovascular treatment. This observation could be attributed to the fact that the fibrous cap on the plaque surface is more vulnerable to damage after EG destruction, leading to plaque instability. HA plays a crucial role in many EG functions, and accelerated HA turnover could be beneficial in maintaining BBB integrity [35]. The degradation of HA largely depends on hyaluronidase activity. HA promotes atherosclerosis by recruiting monocytes and macrophages [36]. HA is highly upregulated in atherosclerotic‐prone areas and alters the stability of atherosclerotic plaques by modulating phenotypic changes in smooth muscle cells within the plaques. Studies have also reported a rich presence of HA in the lumen portion of vulnerable plaques [37]. Notably, plasma HA showed a positive correlation with fibrinogen, hsCRP, HbA1C, ApoB, and blood glucose. Multivariate logistic regression analysis revealed that elevated fibrinogen and HbA1C were influential factors for HA elevation. The EG thickness in diabetic patients is reported to be half that of healthy controls [38], suggesting that chronic hyperglycemia may impair EG by acting on HA. Fibrinogen is an acute phase protein that increases during inflammation [39]. Elevated plasma fibrinogen concentrations can directly activate various mechanisms that might exacerbate atherosclerosis progression [40]. A study on COVID‐19 showed a higher peripheral blood fibrinogen content in patients with elevated plasma HA levels, with correlation analysis demonstrating that fibrinogen content increased with the rise in blood HA concentration [41].

## 5 Conclusion

We determined the levels of plasma glycocalyx molecules in patients with ICAS, proposed the predictive function of HA in the occurrence of adverse cerebral infarction events after ICAS, and preliminarily investigated the influencing factors of peripheral circulation HA changes. Consequently, preserving and restoring the endothelial glycocalyx could potentially be an effective therapeutic target for preventing cerebral infarctions in patients with ICAS after intravascular therapy.

## Author Contributions

Fangfang Zhao wrote the manuscript. Tao Wang and Jichang Luo collect clinical data and organize it. Haoyuan Gao transferred to collect clinical data. Yumin Luo and Liqun Jiao designed and critically revised the manuscript.

## Conflicts of Interest

The authors declare no conflicts of interest.

## Data Availability

The data that support the findings of this study are available upon request from the corresponding author. The data are not publicly available due to privacy or ethical restrictions.
